# Proteins regulating the intercellular transfer and function of P-glycoprotein in multidrug-resistant cancer

**DOI:** 10.3332/ecancer.2017.768

**Published:** 2017-09-18

**Authors:** Deep Pokharel, Ariane Roseblade, Vici Oenarto, Jamie F Lu, Mary Bebawy

**Affiliations:** 1 Discipline of Pharmacy, The Graduate School of Health, The University of Technology Sydney, Sydney, NSW 2007, Australia; 2 Laboratory of Cancer Cell Biology and Therapeutics, The University of Technology Sydney, Sydney, NSW 2007, Australia

**Keywords:** cancer, extracellular vesicles, FERM, microparticles, multidrug resistance, P-glycoprotein

## Abstract

Chemotherapy is an essential part of anticancer treatment. However, the overexpression of P-glycoprotein (P-gp) and the subsequent emergence of multidrug resistance (MDR) hampers successful treatment clinically. P-gp is a multidrug efflux transporter that functions to protect cells from xenobiotics by exporting them out from the plasma membrane to the extracellular space. P-gp inhibitors have been developed in an attempt to overcome P-gp-mediated MDR; however, lack of specificity and dose limiting toxicity have limited their effectiveness clinically. Recent studies report on accessory proteins that either directly or indirectly regulate P-gp expression and function and which are necessary for the establishment of the functional phenotype in cancer cells. This review discusses the role of these proteins, some of which have been recently proposed to comprise an interactive complex, and discusses their contribution towards MDR. We also discuss the role of other pathways and proteins in regulating P-gp expression in cells. The potential for these proteins as novel therapeutic targets provides new opportunities to circumvent MDR clinically.

## Introduction

Multidrug resistance (MDR) is a unique type of resistance by which cancer cells develop cross resistance to a broad spectrum of anticancer agents [1]. It was first described in 1970 as “an altered qualitative difference in cell membranes that resulted in decreased permeability towards actinomycin D and other compounds” [1]. Since this observation, the pathways contributing to drug resistance have been shown to be heterogeneous, dynamic, and persist to confound cancer treatment [1, 2]. The cellular mechanisms contributing to drug resistance are multifactorial and include the overexpression of drug efflux pumps, reduced drug uptake, activation of drug detoxifying systems, drug sequestration and altered drug targets among others [3, 4]. Other prominent mechanisms, by which cancer cells resist the cytotoxic effects of chemotherapeutic drugs, include but are not limited to the following:

### DNA repair mechanisms

Several mechanisms for DNA repair exist, which contribute to resistance to alkylating agents, anthracyclines and platinum compounds [5, 6]. Revision repair by O-6-methylguanine-DNA methyltransferase (MGMT) and nucleotide excision repair are the main mechanisms associated with clinically significant resistance to methylating agents and platinum-containing agents, respectively [7]. MGMT is a protein responsible for the repair of damage to DNA caused by alkylating agents. The overexpression of MGMT in glioblastoma is associated with resistance to alkylating agents, by removing alkyl groups from the O6 position of guanine in tumour cells [8]. The methylation of the promoter region of MGMT gene suppresses MGMT expression, resulting in an improvement in the prognosis of glioma patients [9].

### Autophagy

Autophagy is mediated by a unique organelle called the autophagosome [10]. This is a mechanism of cellular repair, whereby damaged cellular organelles are packaged inside vesicles and delivered to the lysosomes for degradation [11]. Lysosomal degradation results in the generation of cellular energy, which promotes cell survival following episodes of cellular stress such as exposure to chemotherapy, hypoxia or nutrient deprivation [12]. The process of autophagy involves multiple steps including the formation of a vesicular membrane, vesicle nucleation/elongation, docking/fusion of vesicles to lysosomes, and degradation/recycling of the content [13]. The induction of autophagy has been associated with resistance to various drugs, including epirubicin, bevacizumab, sorafenib, gefitinib, erlotinib, used in the treatment of breast cancer, hepatocellular carcinoma, oesophageal carcinoma, lung cancer, glioblastoma, nasopharyngeal cancer, ovarian cancer, prostate cancer, pancreatic adenocarcinoma, and lymphoma [11, 14–21].

### Defective apoptosis

Apoptosis is a highly conserved process of programmed cell death, which is activated by most chemotherapeutic drugs [22], and the dysregulation of which leads to drug resistance and cell survival [7, 23–25]. p53 is a key regulator of the anti-apoptotic B-cell lymphoma 2 (Bcl-2) protein [26]. Mutations to p53 result in the loss of pro-apoptotic function of wild-type p53 and a marked increase in Bcl-2 expression [26, 27], resulting in cancer development, progression, and resistance to chemotherapy [28].

### Tumour microenvironment

The tumour microenvironment also supports resistance to chemotherapeutic drugs. Proteins belonging to the extracellular matrix play an important role in the regulation of cell proliferation, differentiation, and metastasis [29]. For instance, the recruitment of TH2-type tumour-associated macrophages (TAMS) facilitate cancer survival through their supportive role in angiogenesis and in suppressing the CD8+ T-cell antitumour immune response [30]. Likewise the interaction between fibroblasts and cancer cells results in the expression of monocyte colony-stimulating factors, stromal cell-derived factor 1 and matrix metalloproteinases, which are associated with fibroblast-activated cancer and invasive disease [31]. Fibroblasts promote rapid growth of oestrogen receptor-positive breast cancer cells, leading to resistance to those drugs targeting oestrogen receptors, such as tamoxifen [32].

## P-glycoprotein: a member of ATP-binding cassette superfamily of membrane transporters

Membrane transporters play a central role in the emergence of MDR and in the translocation of chemotherapeutics and other substances across the cancer cell plasma membranes. These transporters, physiologically present in most cells, are involved in the efflux of compounds such as lipids, linear, and cyclic peptides, as well as cytotoxic and non-toxic drugs [33]. ABC transporters use ATP hydrolysis to translocate substrates across the cell membrane to the cell exterior [34]. Structural characteristics typical of members of this superfamily include two cytoplasmic nucleotide-binding domains (NBD’s) that contain a conserved sequence motif (the Walker A motif) for the binding and hydrolysis of ATP and two trans-membrane domains (TMD’s) involved in substrate recognition and binding [35–37]. There are 49 ABC genes that are arranged in seven subfamilies of A to G [38, 39]. Currently, the most studied ABC transporters contributing to MDR in cancer are P-gp/*ABCB1* and multidrug-resistant protein 1 (MRP1/*ABCC1*). These transporters are inherently expressed in most normal mammalian cells and are typically localised at sites of xenobiotic exposure [40]. These transporters are found in high levels in the liver, jejunum, blood–brain barrier and kidney and play a significant role in regulating the absorption, distribution, metabolism and ultimately, elimination of xenobiotics across these pharmacological barriers [41, 42].

**Figure 1.**
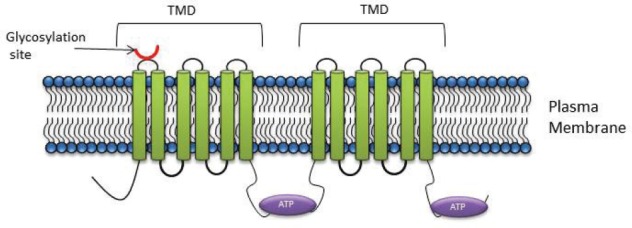
P-gp structure: The core structure of P-gp includes two homologous halves, each half comprising of six transmembrane domains with a nucleotide binding domain.

P-gp is a 170 kDa protein, comprised of a single polypeptide of two homologous halves [43, 44]. Each half comprises of six TMD’s with a NBD on the cytoplasmic side of a membrane [44, 45] (Figure 1). The protective role that P-gp serves in preventing xenobiotic exposure of vital organs has been validated both in studies using MDR knock-out mice and in studies using pharmacological inhibitors [45]. Malignant tumour cells effectively exploit the protective function of members of this superfamily and when hyperexpressed in malignant cells, serve to prevent the accumulation of cytotoxins within the tumour mass.

The first human ABC-transporter cloned, P-gp (*ABCB1*, MDR1) is the most extensively studied member of this superfamily and is regarded as the ‘classical’ ABC-transporter. First isolated from MDR Chinese hamster ovary cells, it was the first candidate associated with an intrinsic role in conferring cancer MDR [46]. The ‘classical’ MDR phenotype, however, was later characterised as a cross-resistance pattern towards vinca alkyloids, anthracyclines, taxanes, and epipodophyllotoxins [42, 47].

## Role of extracellular vesicles in transferring P-glycoprotein

**Figure 2.**
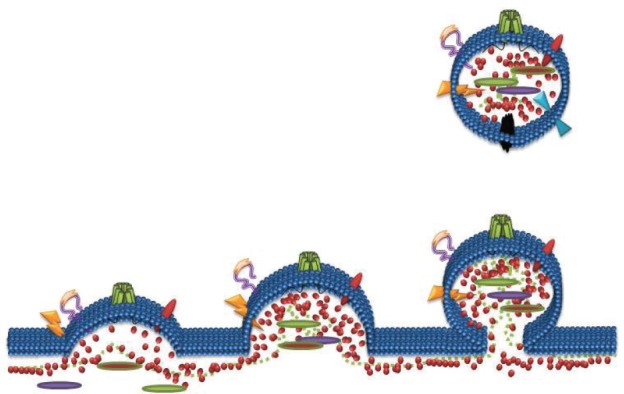
Formation of Microparticles (MPs): MPs are membrane vesicles released from membrane budding following loss of phospholipid asymmetry and cleavage of the underlying cytoskeleton. MPs package and transfer bioactive lipids, nucleic acids and proteins from the parental cells to recipient cells as part of cell-to-cell communication.

Microparticles (MPs) are small membrane extracellular vesicles typically 0.1–1 µm in diameter [48]. MPs are released by various cell types and are involved in many physiological functions such as inflammation, homeostasis, coagulation, chemotherapeutic drug resistance and metastasis [2, 4, 34, 49, 50, 51–55]. MPs are established vectors for the cell-to-cell transfer of bioactive molecules including proteins and nucleic acids from their originating cell and mediate intercellular cross talk by transferring functional proteins, nucleic acids, lipids, antigens and cytokines from donor cells to recipient cells [48, 55–57]. It is not surprising that malignant cells shed a significantly greater number of MP’s in contrast to non-malignant cells [2, 58]. Hence, in cancer patients, the number of circulating MPs as well as their cargo are considerably distinguishable from a healthy patient [52, 53]. We were the first to discover a non-genetic pathway contributing to MDR via MP-mediated transfer of functional resistance proteins and nucleic acids from MDR cells to drug-sensitive cells [48, 55, 59] (Figure 2). Specifically, we showed that MPs were spontaneously shed from P-gp and MRP-1 overexpressed MDR leukaemic/breast cancer cells and contain significant amounts of functional resistance proteins (P-gp and MRP1), together with numerous other proteins and nucleic acids that can establish a functional MDR phenotype, increased metastatic capacity and alter the biomechanical properties of recipient cells [47, 55, 56, 59, 60–63]. In defining the vesicular transfer of MDR, we performed a series of experiments including proteomic profiling and comparative analysis of the MP cargo isolated from MDR and drug-sensitive breast cancer cells [62]. We observed the selective packaging of P-gp, CD44, Ezrin, Radixin, and Moesin in MDR breast cancer derived MPs together with 117 other proteins unique to the resistance cargo, which we proposed may play a role in establishing the MDR phenotype in cancer cell populations [62]. We discuss the role of some of these proteins in this context.

### Tubulins/microtubules

On the basis of its primary amino acid sequence, P-gp consists of two identical domains, each comprised of six transmembrane helices with a large cytoplasmic domain containing the ATP-binding sequences (Figure 1). These two halves are linked by ~90 amino acids, termed the linker domain [64]. Proteins with masses of ~80, 57, and 27 kDa are known to interact with the linker domain [64]. These proteins included α- and β-tubulins [64], emphasising the intimate association between the cytoskeleton and P-gp. Furthermore, the phosphorylation state of P-gp plays an important role in the binding P-gp to tubulin or microtubule filaments [64].

Microtubules are important cellular components that participate in the maintenance of cell morphology and perform cellular functions such as cytokinesis, mitosis/meiosis, secretion, transmembrane signalling, and intracellular transport. Typical substrates of P-gp including vinblastine and vincristine act to destabilise microtubule formation whereas others, including paclitaxel (taxol), docetaxel, and epothilone act to stabilise [65].

Tubulin is found in high levels in drug-resistant cells [66, 67]. Specifically, paclitaxel-resistant murine cells, human lung, ovarian, prostate and breast cancer have increased expression of βI-tubulin [68, 69]. A correlation between βII-tubulin expression was reported with lower docetaxel response rates [70]. An increase in the expression of βIII-tubulin was associated with paclitaxel resistance and decreased expression was observed when cells acquired resistance to vinca alkaloids, including vinflunine [66, 71–73]. Higher levels of βIII-tubulin along with βI-tubulin have also been associated with resistance to taxanes and docetaxel-based chemotherapy in various human cancers [74–76]. The knockdown of βIII-tubulin corresponds with increased sensitivity to tubulin-binding agents and increased sensitivity to DNA-damaging agents [77]. Similar results were reported by Kyu-Ho Han *et al*., who showed that the reduction of α-tubulin by kα1 antisense results in H460/T800 cells becoming more sensitive to the antimitotic drugs including paclitaxel, colchicine, and vinblastine [78]. This may occur through βIII-tubulin effects on increasing microtubule dynamics [79] or by rendering microtubules less sensitive to the effects of paclitaxel [80].

### Ezrin, Radixin, Moesin

A group of highly homologous proteins, Ezrin, Radixin and Moesin, collectively referred to as ERM, belong to the FERM (four-point-one band ERM) domain proteins [81]. These proteins are ~ 300 amino acids long, with a molecular weight of ~ 80 kDa. ERM proteins function as cytoskeletal linkers and are involved in many cellular processes, including migration, growth and adhesion [82]. These functions are facilitated through the binding of ERM proteins directly to the C-terminal domain of actin filaments and to the cytoplasmic NH2-terminal domain of integral membrane proteins such as CD44, intercellular adhesion molecule-1 (ICAM-1), ICAM-2, and CD43 [83]. Structurally, ERM proteins comprise three domains including (1) N-terminal FERM domain, (2) central α-helical domain, and (3) C-terminal tail domain. P-gp has been shown to bind to amino acid residues 149-242 of the N-terminal domain of Ezrin and this binding has been shown to contribute to the P-gp-mediated resistance phenotype [84, 85].

ERM proteins are essential for the stability and functionality of P-gp in normal tissues and in cancer cells [86–89]. Kobori *et al*., reported that anticancer substrates of P-gp activate the Ras homologue gene family member A (RhoA) and Rho-associated protein kinases (ROCK) that increases the expression of ERM, leading to an increase in P-gp expression [89]. Kano *et al*., investigated the functional role of ERM proteins as transcriptional regulators of P-gp following knockdown of individual proteins of the ERM family [85]. Their results showed that ezrin influences the expression of P-gp whereas Radixin reduced P-gp expression by 70% [86]. Similar results were observed by Yang *et al*., in radixin and ezrin silenced Caco-2 cells, where P-gp expression was reduced in both cases [90]. Furthermore, in Caco-2 cell lines, the plasma membrane localisation of P-gp was significantly decreased after knockdown of radixin [90]. In separate experiments, radixin was reported to increase P-gp expression in the small intestine after repeated oral treatment with etoposide [91]. Moesin on the other hand has been shown to have no effect on P-gp expression, but it is often linked with increased tumour size and invasion of cancer cells [92].

### Hyaluronan and CD44

Hyaluronan (HA) is a non-sulphated linear glycosaminoglycan, consisting of repeating disaccharide units of D-glucuronic acid and N-acetyl-D-glucosamine [93]. HA is synthesised at the plasma membrane and released immediately onto the cell surface or into the extracellular matrix [94]. HA interacts with the cell surface in at least two ways, (i) localising at the cell surface by sustained transmembrane interactions with its synthases [94] and (ii) through binding to specific cell-surface receptors such as CD44 and CD168 (RHAMM) [95]. Binding of HA to the N-terminal part of CD44 receptor is involved in resistance to chemotherapeutic drugs in many cancers [96–99]. This is believed to be mediated through effects downstream via epidermal growth factor receptor (EGFR) signalling [96], promoting c-Jun signalling [100], through the interaction with several cytoskeletal proteins [101] and RhoA signalling [101, 102]. Bourguignon *et al*., further validated the effect of RhoA signalling where they demonstrated that HA-induced CD44 interaction with c-Src-activated-Twist plays a major role in microRNA-10b production, leading to downregulation of tumour supressor proteins, RhoA/RhoC upregulation, activation of Rho-kinase and breast tumour cell invasion [103]. Recently, it was shown that inhibiting MDR inhibits the synthesis and secretion of HA, suggesting that HA might be secreted through efflx transporters [104]. We have also shown that vesicles from MDR cells also selectively package HA [105].

CD44 is a transmembrane cell surface glycoprotein, expressed by a large number of cell types and has a critical role in cell proliferation, differentiation, migration and adhesion [106]. The cytoplasmic region of CD44 comprises 72 amino acid residues that have been shown to interact with actin filaments through ERM proteins [107]. The N-terminus of activated ERM proteins bind to a motif between the transmembrane region and the ankyrin-binding site of CD44, and the carboxyl terminus binds to filamentous actin (F-actin), thus linking CD44 to the actin cytoskeleton [107]. P-gp and CD44 are colocalised, coregulated, and coimmunoprecipitated in MDR cells [108]. The introduction of CD44 in cells increases the expression of P-gp within the same cells and knockdown of CD44 highly affects the drug efflux mechanism of P-gp-mediated MDR [81, 108, 109]. Miletti-Gonzalez *et al*., showed that P-gp expression in drug-resistant cells had a positive correlation with the level of CD44 expression [110]. Our laboratory investigated the role of CD44, ezrin, radixin, and moesin in the regulation of P-gp functionality and acquisition of MDR. By sequentially silencing ERM and CD44 proteins in resistant breast cancer cells, we showed that CD44 and radixin, in particular, were required for P-gp-mediated drug efflux functionality, whereas all ERM proteins play a significant role in the vesicular transfer of functional P-gp to the recipient cells [81].

### CD147/Basigin

Matrix metalloproteinases (MMPs) are zinc-dependent endopeptidases that are highly expressed during tumour invasion and metastasis [111]. The increased expression and activity of MMPs in MDR cancer cells is often attributed to the overexpression of CD147 (Basigin), an extracellular MMP inducer [111]. CD147 is a cell-surface glycoprotein that regulates the expression and function of both P-gp and MMPs [112]. Similar to P-gp, CD147 is highly glycosylated, which is one of the many post-translational modifications of proteins closely associated with adhesion, invasion and metastasis of tumour cells [113]. The N-glycosylation of CD147 facilitates its interaction with other proteins [113]. In addition, inhibition of N-glycosylation has been shown to increase the ubiquitination process that degrades both P-gp and CD147 [114]. CD147 is also involved in increasing resistance to P-gp drug substrates and regulates the expression of *ABCB1*, MMP2, and MMP9 via an Erk1/2-dependent signalling pathway in breast cancer cells [115]. CD147 and P-gp coimmunoprecipitate and colocalise in MCF7/Adr cells, and furthermore, after treating these cells with P-gp substrates, CD147 expression is induced [114]. Silencing CD147 gene expression increases chemosensitivity in a human ovarian cancer cell line and knockdown of CD147 in MCF7/Adr cells reduces resistance to P-gp substrate drugs [115, 116].

**Figure 3.**
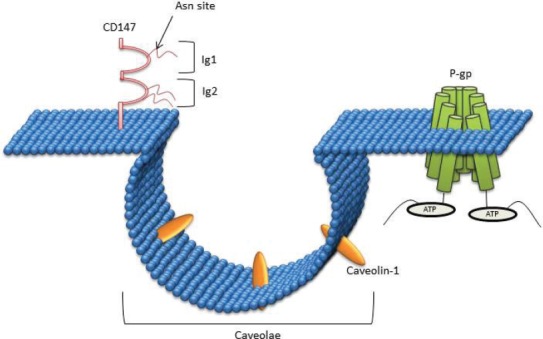
CD147 interaction with Caveolin-1: CD147 is comprised of two immunoglobulin (Ig) domains in the extracellular region, which contain three Asn-linked glycosylation sites. The first Ig domain is involved in MMP induction, whereas the second Ig domain is required for association with caveolin-1.

CD147 is composed of two immunoglobulin (Ig) domains in the extracellular region [117], which contains three Asn-linked glycosylation sites [118]. The first Ig domain is involved in MMP induction whereas the second Ig domain is required for association with caveolin-1 [118] (Figure 3).

### Caveolin

CD147 associates with caveolin (Cav), which is the structural protein of caveolae (small invagination of the plasma membrane (Figure 3)) that interact and regulate the function of a wide variety of proteins known to be involved in homoeostasis, cell proliferation, and adhesion [119]. These 20–24 kDa proteins are highly conserved with three genes expressed in mammalian cells; Cav-1, Cav-2, and Cav-3. Cav-1 and Cav-2 are overexpressed in adipocytes, endothelial cells, and fibroblasts, whereas Cav-3 is explicitly expressed in striated muscle cells [120]. In human cells, Cav-1 has two isoforms; the α-isoform (178 amino acids) and β-isoform (32 amino acids).

The upregulation of Cav-1 in MDR cells occurs upon short exposure to cytotoxic drugs [123, 122]. This upregulation is observed at both RNA and the protein level after 24 h of cytotoxin exposure [121, 122]. Similarly, P-gp localises in the caveolae of MDR cells [121] where it is colocalised with Cav-1 in many cells including CHRC5 chemoresistant cells [123], A549 lung carcinoma cells [124] and brain endothelial cells [123]. The downregulation of Cav-1 increases P-gp functionality [125]. The high expression of Cav-1 has been identified in a number of MDR cancer cells, including colchicine-resistant HT-29-MDR cells [126], vinblastine-resistant SKVLB1 ovarian carcinoma cells [121], taxol-resistant A549-T24 lung carcinoma cells [121], and adriamycin-resistant MCF-7 breast adenocarcinoma cells [127].

### Rab-related proteins

P-gp is primarily localised in the plasma membrane, but it is also localised intracellularly in the endoplasmic reticulum, golgi, endosome, lysosome and proteasome [127–130]. These intracellular sites are important in the synthesis, post-translational modification, traffic/recycling, and degradation of P-gp [128–131]. P-gp trafficking and recycling is performed with a group of proteins commonly known as Rab proteins [132]. Rab proteins are part of the Ras superfamily of small GTPases known to regulate most vesicular transport events by regulating vesicle docking and fusion [133, 134]. There are more than 60 Rab proteins in mammalian cells and most of them are localised in the subcellular membrane compartment, where they control the intracellular trafficking routes of proteins and lipids [135, 136]. These proteins are often associated with P-gp. Involvement of Rab6 is observed during the trafficking of P-gp from Golgi to the plasma membrane [132], whereas Rab11 and Rab13 are involved in the trafficking of P-gp from Golgi to the recycling endosome [137, 138] and Rab11a has been shown to be involved in the trafficking P-gp to the apical membrane in polarised cells [139]. The overexpression of Rab4 in MDR cells is associated with an increase in drug sensitivity through Rab4-mediated localisation of P-gp in cytosolic endosomal compartments [140]. Rab4 along with Rab14 are involved in membrane protein trafficking and interact with the C-terminal of P-gp [140]. Rab4 co-localises with P-gp in the cytoplasmic compartments [140]. Similar results were observed with the overexpression of Rab6c protein, which resulted in the intracellular localisation of P-gp and increased accumulation of anticancer drugs [141]. Similarly, transfection of dominant negative Rab5 increased the intracellular localisation of P-gp by 9-fold and consequently increases the intracellular accumulation of daunorubicin. These studies support strategies for the circumvention of MDR through the regulation of subcellular redistribution of P-gp [142]. Table 1 summarises the role of Rab proteins in cancer.

### Ubiquitination

Ubiquitination is a process that regulates P-gp expression. It is a reaction where ubiquitin molecules are covalently ligated to substrate proteins via isopeptide bonds formed through the C-terminal glycine to the ε-amino group of lysine residues [143]. This reaction plays an important role in cell-surface signalling as well as in endocytosis and degradation of ATP-binding cassette transporters [144, 145]. Ubiquitin consists of a conserved 76-amino acid polypeptide and the conjugation of ubiquitin to substrates is a three step process involving: activation by ubiquitin-activating enzyme (E1), conjugation by ubiquitin-conjugating enzymes (E2) and ligation by ubiquitin–ligase enzyme (E3) [146].

Several transcription factors including c-Jun, FOXO3a, the vitamin D receptor and nuclear factor-κB (NF-κB) are involved in the transcription of *ABCB1* [147–150]. These transcription factors are regulated by ubiquitination, highlighting the association between ubiquitin and drug resistance. For example, ubiquitin promotes the binding of c-Jun to the activator protein-1 (AP-1) site of the *ABCB1* promoter to reduce transcription [149]. FBXO15/FBX15 (F-box proteins and a part of the Skp1-Cullin1-FBXO15 ubiquitin E3 ligase complex) and Ube2rl/Cdc34/Ubc3 (E2 enzymes) regulate P-gp expression through effects on the ubiquitin–proteasome pathway [131]. Similarly, the activation of JNK pathway via E3 ubiquitin ligase downregulates P-gp transcription [151]. Recent studies have also shown that inhibition of the PI3K/Akt-signalling pathway can lead to decreased cell growth, tumour formation, and reverse P-gp-mediated MDR [61]. Interestingly, a casitas B-lineage lymphoma-b (Cbl-b) protein, which is a member of the E3 ubiquitin ligase family, interacts with the p53-regulatory subunit of PI3K and results in PI3K ubiquitination and degradation [152]. Nonetheless, transfection of MDR cells with wild-type ubiquitin or treatment with an N-glycosylation inhibitor increases the ubiquitination of P-gp and increases P-gp degradation [143, 144].

### Heat-shock proteins

Heat-shock proteins (Hsps) are molecular chaperones that facilitate folding of newly synthesised polypeptides, stabilisation, refolding and protein trafficking [154] and are involved in regulating P-gp expression. They are classified based on their molecular weights: hsp100, 90, 70, 60, 40 and ‘small hsps’ [154]. Hsp proteins such as 27, 40, and 70 play an important role in the cell before the unfolded protein reaches the hsp90 [155]. These Hsp proteins facilitate rapid cell division, metastasis, and evasion of apoptosis in cancer cells [154]. They are generally expressed in all cell types, but their synthesis increases during times of cellular, chemical and physical stress, and during exposure to anticancer drugs [154].

Hsp 90 has emerged as a major therapeutic target for cancer therapy because of its ability to bind and stabilise a broad range of proteins [156]. The inhibitor of Hsp90 together with an inhibitor of Sirtuin-1 inhibit the growth of chemoresistant cells isolated from human chronic myeloid leukaemia K562 cells [157]. Similarly, inhibition of Hsp90 leads to the prolonged inhibition of Akt signalling, inactivates NF-κB and eventually causes resistance to chemotherapeutics and molecularly targeted drugs [158]. Nonetheless, activated HSP70 and NF-κB in drug-resistant A549/DOX cells modulate P-gp expression resulting in doxorubicin retention and enhanced apoptosis [159].

### Glutathione S-transferase

Glutathione s-transferase (GST) is a superfamily of dimeric proteins catalysing the conjugation of glutathione with a wide variety of electrophiles [160]. GSTs are involved in development of drug resistance to chemotherapy through direct detoxification and by inhibiting the mitogen-activated protein kinase (MAPK) pathway [161]. According to their amino acid sequences, these families are divided into four multigene classes, α, μ, π, θ (Cullen *et al*., 2003). GST-π located on chromosome 11q13, plays an important role in the detoxification of xenobiotics through conjugation to glutathione [162]. It is often related with MDR because of its characteristic to catalyse the conjugation of glutathione and anticancer drugs and then expel them from cells via GSH-conjugated export pumps [163]. GST-π is expressed at low levels in the placenta, lung, liver, kidney, and red blood cells [164], has been shown to correlate with prognosis and is associated with MDR [165, 166]. The coexpression of P-gp and GST-π was shown to be 93% in patients with leukaemia and 80% in patients with lung cancer [167]. Similar results were reported by another group where high levels of GST-π significantly contributed to clinical cisplatin resistance in different types of human cancers [168].

## Discussion and conclusions

**Figure 4.**
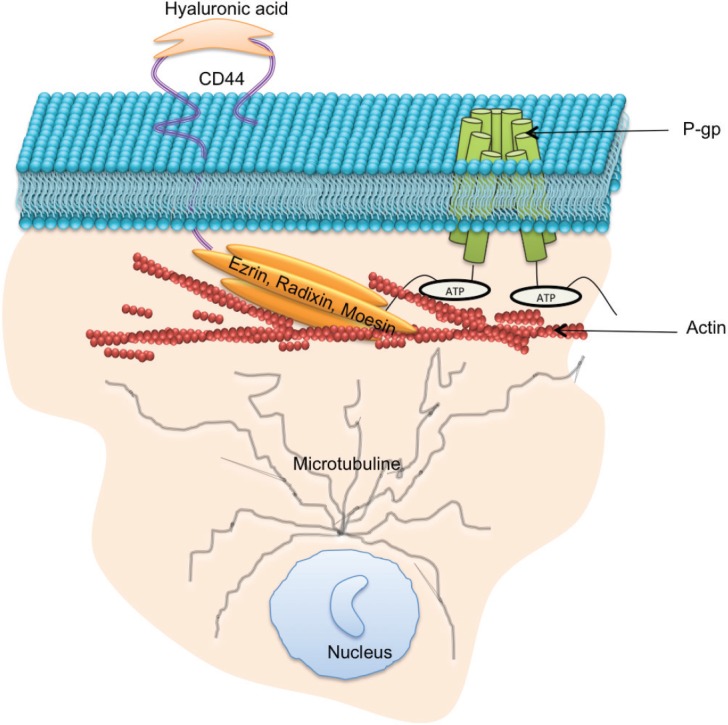
Protein–protein interactions which support P-gp drug efflux function: P-gp drug efflux function and intercellular transfer has been shown to to be regulated by a number proteins including microtubule, ezrin, radixin, moesin, and CD44.

For more than three decades, researchers have searched for effective ways to inhibit P-gp as a way of circumventing MDR in cancer [169]. There are currently four generations of P-gp inhibitors, and few of them have reached clinical trial [170]. These inhibitors have been limited by low binding affinities towards P-gp, requiring their use at high doses and hence resulting in dose-limiting toxicity [159]. P-gp is expressed in numerous cells and tissues where it functions to efflux potentially harmful xenobiotics preventing their accumulation within specialised tissues [171]. The current inhibitor molecules are non-selective and target both the overexpressed P-gp in tumour cells and the endogenous P-gp, resulting in altered pharmacokinetic profiles and increasing risk of drug toxicity. Hence, an alternative approach is required to overcome P-gp-mediated MDR that supports the accumulation of chemotherapeutics drugs within the tumour mass while not compromising the inherent defence mechanism of normal cells. There is much evidence supporting the theory that drug efflux is not solely dependent on P-gp itself but also on proteins it interacts with or which regulate its expression (Figure 4). Our laboratory has previously used gene-silencing strategies targeting ezrin, radixin, moesin and CD44 proteins [81] and observed that CD44 and radixin reduce P-gp functionality to the greatest extent. We also reported that the vesicular transfer and acquisition of P-gp in recipient cell membranes was regulated by the presence of ezrin and moesin. These results demonstrate the tight association between this group of proteins with P-gp and strengthen opportunities for alternative approaches in overcoming MDR. This paper describes numerous proteins that are involved in regulating P-gp-mediated MDR, either directly or indirectly and introduces novel strategies to overcome cancer drug resistance, which can be considered further.

## Figures and Tables

**Table 1.** Rab-related proteins in cancer.

	Name	Function in cancer	References
1	Rab1b, Rab4b, Rab10, Rab22a, Rab24	Overexpressed in hepatocellular carcinoma	[172]
2	Rab1a	Overexpressed in tongue squamous cell carcinoma	[173]
3	Rab2	Overexpressed in peripheral blood mononuclear cells from patients with solid tumour	[174]
4	Rab2	Associated with lung tumour progression in mouse	[175]
5	Rab3B	Upregulated in prostate cancer and promotes cancer cell survival	[176]
6	Rab20	Overexpressed in exocrine pancreatic carcinoma	[177]
7	Rab31	Associated with Breast cancer patients	[178]
8	Rab25	Overexpressed in breast cancer and ovarian cancer and associated with decreased survival	[179]
9	Rab8	Mediates exocytosis of matrix metalloprotease involved in cell invasion	[180]
